# Mathematical Model of the Role of Asymptomatic Infection in Outbreaks of Some Emerging Pathogens

**DOI:** 10.3390/tropicalmed5040184

**Published:** 2020-12-09

**Authors:** Nourridine Siewe, Bradford Greening, Nina H. Fefferman

**Affiliations:** 1 School of Mathematical Sciences, Rochester Institute of Technology, College of Sciences, 1 Lomb Memorial Dr, Rochester, NY 14623, USA; 2 Centers for Disease Control and Prevention, 1600 Clifton Rd NE, Atlanta, GA 30329, USA; bradford.greening@gmail.com; 3 Department of Ecology and Evolutionary Biology, College of Sciences, The University of Tennessee, 447 Hesler, 569 Dabney Hall, 1416 Circle Dr, Knoxville, TN 37996-1610, USA; nina.h.fefferman@gmail.com

**Keywords:** emerging and reemerging disease, asymptomatic infection, disease outbreaks

## Abstract

Preparation for outbreaks of emerging infectious diseases is often predicated on beliefs that we will be able to understand the epidemiological nature of an outbreak early into its inception. However, since many rare emerging diseases exhibit different epidemiological behaviors from outbreak to outbreak, early and accurate estimation of the epidemiological situation may not be straightforward in all cases. Previous studies have proposed considering the role of active asymptomatic infections co-emerging and co-circulating as part of the process of emergence of a novel pathogen. Thus far, consideration of the role of asymptomatic infections in emerging disease dynamics have usually avoided considering some important sets of influences. In this paper, we present and analyze a mathematical model to explore the hypothetical scenario that some (re)emerging diseases may actually be able to maintain stable, endemic circulation successfully in an entirely asymptomatic state. We argue that an understanding of this potential mechanism for diversity in observed epidemiological dynamics may be of considerable importance in understanding and preparing for outbreaks of novel and/or emerging diseases.

## 1. Introduction

Preparing for outbreaks of emerging infectious diseases is one of the great modern challenges in global public health. Such preparation is often predicated on beliefs that we will be able to understand the epidemiological nature of an outbreak early into its inception [1,2,3]. Rapid, early analysis is expected to enable assessment of available interventions and inform effective action plans that minimize societal disruption to the extent possible. Emerging diseases such as SARS and MERS are excellent examples of early interventions limiting the global spread of new cases [4,5]. However, early and accurate estimation of the epidemiological situation may not be so straightforward in all cases. Rare or emerging diseases can exhibit different epidemiological behaviors from outbreak to outbreak, leaving it unclear how to best characterize the relevant facets that could be exploited for outbreak mitigation/control.

Mathematical models frequently assume introduction of a novel pathogen into a population assumed to be entirely susceptible to infection and explore the effects of extrinsic influences on the subsequent epidemiological progression. These influences routinely include characterizing differences in the physical environment (e.g., regional or seasonal climatic factors [6,7,8]), in human social behaviors and therefore contact patterns (e.g., daily opportunities for passive transmission), in access to and practice of medical care (either shifting the initial health and subsequent susceptibility to infection, or altering care of those already infected and therefore changing the trajectory of their illness [9]), or in diversity in the pathogen itself due to parallel invasion by multiple strains [10] or due to ongoing mutations as the pathogen spreads [11]. While each of these are of clear potential importance in shaping the course of an outbreak, there may be an additional (and thus far mostly overlooked) mechanism playing a significant role in the dynamics of emerging infections: the transmission and stable circulation of asymptomatic infections [12,13] in the absence of medically observable or identifiable cases. For example, neglecting asymptomatic carriers in a model of Ebola virus transmission was shown to significantly overestimate the projected cumulative incidence of symptomatic infections [14,15].

Some studies have already proposed considering the role of active asymptomatic infections co-emerging and co-circulating as part of the process of emergence of a novel pathogen. Discussions have generally suggested that asymptomatic vs. clinical outcomes from disease exposure may be due to underlying differences in general health, immunocompetence, or age of the host [6]. Critically, most of these discussions also make the assumption that asymptomatic cases are due to insufficient pathogen replication in the host, and therefore also assume that asymptomatic cases are relatively incapable of transmitting infectious pathogens to others, although some studies have suggested the need for expansion to include asymptomatic transmission [14,15,16,17]. Examples of such studies include multi-strain SEIR epidemic models with general incidence that establish the global stability of disease-free and various endemic equilibrium states [18,19].

Thus far, however, consideration of the role of asymptomatic infections in emerging disease dynamics have usually avoided considering the full set of possible influences. In addition to the simultaneous influence of clinical and asymptomatic cases competing for available susceptible hosts, there is also the possibility that some diseases remain asymptomatic not due to poor relative replication in a host after exposure to an average exposure to an infectious case, but instead that clinical outcomes may be in response to exposure dosage. In this case, it is possible that asymptomatic cases themselves produce doses of infectious exposure that an average immune response can control, but not so quickly as to render the infection truly inert in the host. Note that most epidemiological models that account for asymptomatic individuals assume that this latter human category can transmit the disease, but do not show disease symptoms within the epidemic time frame [20,21]. However, in the case of endemicity such as malaria, asymptomatic carriers were shown to have a major effect on the calculation for the basic reproduction number, as well as in determining the bifurcations that might occur at the onset of disease-free equilibrium [22]. The existence of dose-response based infection is already established for some pathogens [23], therefore it may not be unreasonable to consider that such behaviors may play a critical role in the emergence of some novel pathogens as well.

We propose that some (re)emerging diseases may actually be able to maintain stable, endemic circulation successfully in an entirely asymptomatic state. Circulating asymptomatic cases could potentially provide hosts with immunity (either full or partial) to subsequent infection exposure. For exposure to lead to infection, it may be possible that the cumulative action of relatively rapid re-exposures to asymptomatic carriers could lead to a sufficient dose as to trigger clinical signs and symptoms of infection (just as would be expected from naive exposure to a fully infectious host), or conversely, these doses could be strictly insufficient to ever trigger full infection. The range of potential behaviors in such hypothetical systems are substantially diverse. While there is currently little evidence for the existence/prevalence of such systems, it is also true that they might be prohibitively difficult to detect a priori. By their definition, emerging infectious diseases have not yet been known to circulate widely. Few medical studies have focused on transmission dynamics for microorganisms that are not currently understood to cause disease, unless they are close relatives of existing pathogens (e.g., benign dermal staphylococcus colonization, etc. [24]). It is therefore not unlikely that asymptomatic circulation might play a role in the disease dynamics of emerging infections before we could even detect a first clinical case. This pre-circulation could conceivably affect our ability to quantify transmission (β${}^{\prime }$s) and reproductive capability ($R_{0}$) for outbreaks of novel pathogens, causing fluctuation in estimates attributable to, all other things being equal, whether or not asymptomatic infections had already been circulating unnoticed in the affected populations. This offers a potential alternative explanation for why outbreaks may behave very differently as they leave native ranges, even (or possibly especially) when native-range outbreaks are rare, and therefore are not expected to alter the epidemiological landscape for subsequent outbreaks within the same region.

For these hypothetical cases to be plausible for any emerging infectious disease, we must show that stable endemic circulation for asymptomatic infections is possible without necessarily leading to epidemic spread of clinical infections. Further, it would lend credibility to these scenarios if we could show that rare outbreaks of clinically significant infections do not necessarily cause the population to revert to fully susceptible and could instead allow stable populations of asymptomatic cases to be maintained, even as the clinical infections die out with or without control measures. We here present and analyze a mathematical model to explore the hypothetical existence of just such scenarios and demonstrate the potential importance of asymptomatic infections in shaping the dynamics of emerging infectious diseases. We argue that an understanding of this potential mechanism for diversity in observed epidemiological dynamics may be of considerable importance in understanding and preparing for outbreaks of novel and/or emerging diseases.

## 2. Methods

**Figure 1:**
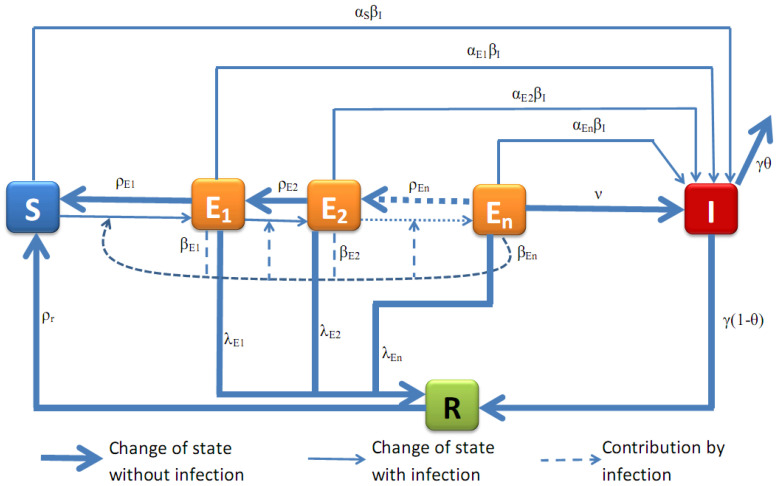
Framework. The nodes with reddish colors (orange and red) represent the compartments of humans that are infectious, while the nodes with greenish colors (black and green) represent the compartments of humans that are not infectious. The various interactions are explained in the descriptions of Equations (1)–(6).

In this section we introduce a mathematical model for a generic viral, contact-transmissible disease that includes different levels of asymptomatic (latent) stages of infection, as shown in Figure 1. The mathematical model, which is compartmental of type SEIR [25,26,27,28,29,30], is based on the framework in Figure 1. Note that this model is easily applicable to or extendable for a wide range of diseases like Ebola Virus Disease (EVD), *Staphylococcus aureus* [14], *Streptococcus pneumoniae* [14], and *Neissera meningitidis* [31].

We denote by *S* the number of humans who have not had any contact with the virus. The compartment *E* is subdivided into several sub-compartments, $E_{i},\;i=1,2,\ldots ,n$, that represent the different levels of asymptomatic transmission. We assume that the force of transmission gets larger as the index i=1,2,…,n, increases; that is the probability of a successful infection after contact of humans of type $E_{i}$ with humans of type *I* is higher as *i* increases. We also assume that there exists $\tilde{n},\;1\leq \tilde{n}<n$, such that $E_{k}$ does not progress to higher levels of infectiousness ($E_{k+1}$, say) whenever $k\leq \tilde{n}$. This $\tilde{n}$ accounts for the load of pathogens in the body that could be considered mild and unable to cause more harm. We denote by *I* the number of infected humans who show clinical signs of the disease, and by *R* the number of humans who recover from the disease.

**Figure 2:**
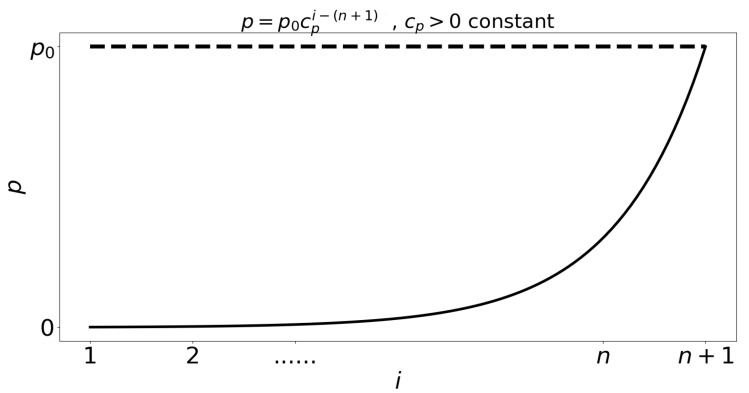
Profile for the parameters $\alpha_{i}$${}^{\prime }$s, $\beta_{i}$${}^{\prime }$s and $\lambda_{i}$${}^{\prime }$s, represented here by $p=p_{0}c_{p}^{i-(n+1)}$, i∈{1,2,…,n}, where $c_{p}>0$ is a constant. More details in Technical Appendix.

Assumptions about the structure of our model include the following:

1. Individuals infected by symptomatic individuals immediately become symptomatic without passing through an asymptomatic stage, whereas individuals infected by asymptomatic individuals become asymptomatic. This assumption is inspired by an exponential shedding dose-response curve, as illustrated in Figure 2.
2. Individuals at earlier asymptomatic stages require further infection events to progress to the next asymptomatic stage, while individuals at later asymptomatic stages can automatically progress to the symptomatic stage.
3. Individuals at earlier asymptomatic stages can only move onto the next asymptomatic stage if infected by those at higher asymptomatic stages of infection, or symptomatic individuals.
4. Asymptomatic individuals can revert to earlier asymptomatic stages, but symptomatic individuals cannot revert to asymptomatic infection.
5. For simplicity, we assume that pathogen mutations are not included, and thus, disease properties such as transmission, aggressivity and mortality remain unchanged in time. We also do not consider the intrinsic potential of the pathogen to lay dormant within the host, and assume that the pathogen is always active and able to infect.

In Figure 1, the nodes with reddish colors (orange and red) represent the compartments of humans that are infectious ($E_{i}$${}^{\prime }$s and *I*), while the nodes with greenish colors (black and green) represent the compartments of humans that are not infectious (*S* and *R*).

### 2.1. Model Presentation

The list of the variables used in our model is presented in Table 1. We now describe the equations for each variable.

Equation for susceptible humans, *S*:

(1) $$\begin{array}{cc} \frac{dS}{dt}= & \;\underset{\mathrm{new}\;\mathrm{recruitment}}{\underset{︸}{b}}+\underset{\mathrm{gain}\;\mathrm{from}\;E_{1}}{\underset{︸}{\rho_{E_{1}}E_{1}}}+\underset{\mathrm{gain}\;\mathrm{from}\;\mathrm{wane}\;\mathrm{of}\;\mathrm{immunity}}{\underset{︸}{\rho_{r}R}}\\ & \;-\underset{\mathrm{loss}\;\mathrm{from}\;E_{i}{}^{\prime }s-\mathrm{infection}}{\underset{︸}{\sum \beta_{E_{i}}E_{i}S}}-\underset{\mathrm{loss}\;\mathrm{to}\;I}{\underset{︸}{\alpha_{S}\beta_{I}SI}}-\underset{\mathrm{natural}\;\mathrm{death}}{\underset{︸}{\mu S.}} \end{array}$$

The first term in the right-hand side of Equation (1) is the gain in *S* as a result of natural birth or human immigration. We assume that all new recruitment in the human population is through the compartment of susceptible humans, *S*. We assume that humans at asymptomatic state $E_{1}$ lose their capacity of infecting humans of type *S*, and return to the compartment *S* after some time. The second term represents the gain in *S* from the transition of asymptomatic compartment $E_{1}$. When a sick person is treated or recovers, he/she may eventually return to the class *S* by waning his/her immunity. This is represented by the third term in the right-hand side of Equation (1). The fourth term is the loss in *S* due to infection by contact with humans of asymptomatic type $E_{i},\;i=1,2,\ldots ,n$. The fifth term is the loss in *S* to *I* due to infection by *I*. We assume, for simplicity, that the baseline infection rate remains constant throughout the year. We also assume that the infection term $\beta_{I}SI$ is weighted by a factor $\alpha_{S}$ that gauges the force of infection; while the last term is the loss in *S* by natural death or immigration.

Equations for exposed humans, $E_{k},\;k=1,2,\ldots ,n$:

(2) $$\begin{array}{cc} \frac{dE_{1}}{dt}= & \;\underset{\mathrm{gain}\;\mathrm{from}\;E_{i}{}^{\prime }s-\mathrm{infection}}{\underset{︸}{\sum \beta_{E_{i}}E_{i}S}}+\underset{\mathrm{gain}\;\mathrm{from}\;E_{2}}{\underset{︸}{\rho_{E_{2}}E_{2}}}-\underset{\mathrm{loss}\;\mathrm{to}\;S}{\underset{︸}{\rho_{E_{1}}E_{1}}}-\underset{\mathrm{loss}\;\mathrm{to}\;E_{2}}{\underset{︸}{\left(\sum_{i>1}\beta_{E_{i}}E_{i}+\nu_{E_{1}}\cdot 0\right)E_{1}}}\\ \; & \;-\underset{\mathrm{loss}\;\mathrm{to}\;I}{\underset{︸}{\alpha_{E_{1}}\beta_{I}E_{1}I}}-\underset{\mathrm{loss}\;\mathrm{to}\;R}{\underset{︸}{\lambda_{E_{1}}E_{1}}}-\underset{\mathrm{natural}\;\mathrm{death}}{\underset{︸}{\mu E_{1}.}} \end{array}$$

We assume that when humans of type *S* are in contact with humans of type $E_{i},\;i=1,2,\ldots ,n$, there may be a change of state from *S* to $E_{1}$. That is, there is a gain in $E_{1}$, only, when *S* gets infected with $E_{i}$${}^{\prime }$s. This is represented by the first term in the right-hand side of Equation (2). The second term is similar to the second term in the right-hand side of Equation (1); that is, the gain in $E_{1}$ due to the transition from $E_{2}$ as a result of the wane of infectious force. The third term is the loss of $E_{1}$ to *S* as described in the second term of Equation (1). We assume that contacts between humans of type $E_{1}$ and humans of type $E_{i},\;i>1$, may lead to the transition to $E_{2}$, only. This is represented by the fourth term in the right-hand side of Equation (2) where the term $\nu_{E_{1}}E_{1}$, the transition from $E_{1}$ to $E_{2}$, is multiplied by zero because we take $\tilde{n}=1$; i.e., individuals with infectious level $E_{1}$ do not progress to $E_{2}$ in absence of contacts with one or other higher sources of infection. Similar to the fifth term in the right-hand side of Equation (1), the fifth term in the right-hand side of Equation (2) represents the loss in $E_{1}$ due to infection by *I*. This term has a weight $\alpha_{E_{1}}$ that gauges the force of transition from $E_{1}$ to *S*. Note that $\alpha_{S}<\alpha_{E_{1}}$. The sixth term is the loss in $E_{1}$ to *R* due to recovery, and the last term in natural death of $E_{1}$.

Recall that there exists $\tilde{n},\;1\leq \tilde{n}<n$, such that $E_{k}$ can “naturally” transit to $E_{k+1}$ when $n>\tilde{n}$, and they do not transit otherwise. Hence, for each intermediate level of asymptomatic class, $E_{i},\;1<i<n$, we have the following equation:

(3) $$\begin{array}{cc} \frac{dE_{k}}{dt} & =\underset{\mathrm{gain}\;\mathrm{from}\;E_{k-1}}{\underset{︸}{\left(\sum_{i>k-1}\beta_{E_{i}}E_{i}+\nu_{E_{k-1}}\right)E_{k-1}}}+\underset{\mathrm{gain}\;\mathrm{from}\;E_{k+1}}{\underset{︸}{\rho_{E_{k+1}}E_{k+1}}}-\underset{\mathrm{loss}\;\mathrm{to}\;E_{k-1}}{\underset{︸}{\rho_{E_{k}}E_{k}}}-\underset{\mathrm{loss}\;\mathrm{to}\;I}{\underset{︸}{\alpha_{E_{k}}\beta_{I}E_{k}I}}\\ & \;-\underset{\mathrm{loss}\;\mathrm{to}\;E_{k+1}}{\underset{︸}{\left(\sum_{i>k}\beta_{E_{i}}E_{i}+\nu_{E_{k}}\cdot \chi_{\tilde{n}}\left(k\right)\right)E_{k}}}-\underset{\mathrm{loss}\;\mathrm{to}\;R}{\underset{︸}{\lambda_{E_{k}}E_{k}}}-\underset{\mathrm{natural}\;\mathrm{death}}{\underset{︸}{\mu E_{k}}},\;k=2,\ldots ,n-1; \end{array}$$

where $\chi_{\tilde{n}}\left(k\right)$ is a choice function that takes value 1 when $k>\tilde{n}$ and 0 otherwise. The terms in the right-hand side of Equation (3) are similar to the terms in the right-hand side of Equation (2) up to respective indexes, except the first terms which are different, though having the same fundamental meaning. The difference lies in the fact that in Equation (2), the first term includes contacts of all humans of asymptomatic classes with *S*; while the first term in Equation (3) includes contacts of all humans of asymptomatic classes with indexes greater than or equal to the current asymptomatic index class (*k*) with the asymptomatic class of preceding index (k−1). The last term is the natural death of $E_{k},\;1<k<n$.

We now write the equation for the highest level of asymptomatic class of humans.

(4) $$\begin{array}{cc} \frac{dE_{n}}{dt}= & \;\underset{\mathrm{gain}\;\mathrm{from}\;E_{n-1}}{\underset{︸}{\left(\beta_{E_{n}}E_{n}+\nu_{E_{n-1}}\right)E_{n-1}}}-\underset{\mathrm{loss}\;\mathrm{to}\;I}{\underset{︸}{\alpha_{E_{n}}\beta_{I}E_{n}I}}-\underset{\mathrm{loss}\;\mathrm{to}\;E_{n-1}}{\underset{︸}{\rho_{E_{n}}E_{n}}}-\underset{\mathrm{fraction}\;\mathrm{that}\;\mathrm{show}\;\mathrm{signs}}{\underset{︸}{\nu_{E_{n}}E_{n}}}\\ \; & \;-\underset{\mathrm{loss}\;\mathrm{to}\;R}{\underset{︸}{\lambda_{E_{n}}E_{n}}}-\underset{\mathrm{natural}\;\mathrm{death}}{\underset{︸}{\mu E_{n}.}} \end{array}$$

The fourth term in the right-hand side of Equation (4) is the loss in $E_{n}$ to *I* due to clinical manifestation of the disease. The remaining terms are similar to those in Equations (2) and (4)

Equation for infectious humans that show clinical signs, *I*:

(5) $$\begin{array}{cc} \frac{dI}{dt}= & \;\underset{\mathrm{gain}\;\mathrm{from}\;S\;\mathrm{and}\;E_{i}{}^{\prime }s}{\underset{︸}{\beta_{I}\left(\alpha_{S}S+\sum \alpha_{E_{i}}E_{i}\right)I}}+\underset{\mathrm{fraction}\;\mathrm{of}\;E_{n}\;\mathrm{that}\;\mathrm{show}\;\mathrm{signs}}{\underset{︸}{\nu_{E_{n}}E_{n}}}-\underset{\mathrm{disease}-\mathrm{induced}\;\mathrm{death}}{\underset{︸}{\gamma \theta I}}\\ \; & \;-\underset{\mathrm{loss}\;\mathrm{from}\;\mathrm{immunization}}{\underset{︸}{\gamma (1-\theta )I}} \end{array}$$

The first term in the right-hand side of Equation (5) is the gain in *I* due to infection of *S* and the $E_{i}$${}^{\prime }$s by contact with *I*. The second term in the gain in *I* as $E_{n}$ show clinical signs of the disease. The third term is the disease–induced death, while the last term is the loss as sick humans recover from the disease. We assume that the time it takes for a sick person to die of, or recover from, the disease is short enough so that we could neglect it, relative to the life expectancy of the individual under study. As such, we drop the term for natural death of the sick humans.

Equation for recovered humans, *R*:

(6) $$\begin{array}{cc} \frac{dR}{dt} & =\underset{\mathrm{gain}\;\mathrm{from}\;\mathrm{immunization}}{\underset{︸}{\gamma (1-\theta )I}}+\underset{\mathrm{gain}\;\mathrm{from}\;\mathrm{immunization}}{\underset{︸}{\sum \lambda_{E_{i}}E_{i}}}-\underset{\mathrm{loss}\;\mathrm{to}\;S}{\underset{︸}{\rho_{r}R}}-\underset{\mathrm{natural}\;\mathrm{death}}{\underset{︸}{\mu R.}} \end{array}$$

The first term in the right-hand side of Equation (6) is the gain in *R* as *I* recovers. The second term is the recovery of asymptomatic humans. The third term is the loss in *R* as recovered humans wane their immunity, while the last term in the natural death of *R*.

The initial conditions for System (1)–(6) are such that

(7) $$\begin{array}{cc} S(0)> & \;0,\;I\left(0\right)\geq 0,\;R\left(0\right)\geq 0,\;E_{i}\left(0\right)\geq 0,\;i=1,2,\ldots ,n,\\ \mathrm{where} & \;E_{j}\left(0\right)>0\;\mathrm{for}\;\mathrm{some}\;j>\tilde{n}. \end{array}$$

At least one set of initial conditions in Equation (7) holds whenever infection occurs in a community. Theorem 1 in [32] establishes that our model is well–posed. The values of the parameters that are used subsequently are all starting values for demonstrating possible disease dynamics; these values are further varied to show the effects of each parameter (Appendix A).

### 2.2. Numerical Results

We simulate Model (1)–(6) where n=6 and $\tilde{n}=1$. The values of the parameters are as in Table 2 and the initial conditions are given by

(8) $$\begin{array}{cc} S\left(0\right)=N_{0}, & \;E_{1}\left(0\right)=1,\;E_{i}\left(0\right)=0,\;i\in \left\{2,3,4,5,6\right\},\;I\left(0\right)=R\left(0\right)=0;\;\mathrm{or} \end{array}$$

(9) $$\begin{array}{cc} S\left(0\right)=N_{0}, & \;E_{1}\left(0\right)=0,\;E_{2}\left(0\right)=1,\;E_{i}\left(0\right)=0,\;i\in \left\{3,4,5,6\right\},\;I\left(0\right)=R\left(0\right)=0. \end{array}$$

The equations are reproduced for n=3 and $\tilde{n}=1$ in Appendix A.1, and the basic reproduction number, $R_{0}$, is calculated for this system of equations.

We compute the ‘outbreak relative severity’ (ORS) as the ratio of the total number of new clinical cases to the total number of new asymptomatic cases, and write:

(10) $$\begin{array}{c} ORS=\frac{\int_{t=0}^{T}\left[Newclinicalcasesattimet\right]dt}{\int_{t=0}^{T}\left(\sum_{i=1}^{6}\left[Newasymptomaticcasesattimet\right]\right)dt}, \end{array}$$

where *T* is the observation time. ORS measures how many clinical cases have been recorded throughout the outbreak as compared to the number of asymptomatic cases. Since the actual total number of asymptomatic cases is usually not known, the ORS as derived by this model can serve as an estimation for this number.

**Figure 3:**
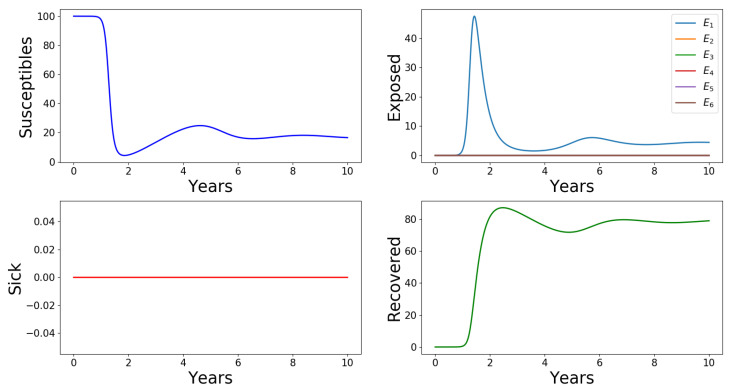
Profiles for all the variables of Model (1)–(6). The horizontal axes scale the time in days and the vertical axes scale the population sizes percentages. The first infectious case arises in the population $E_{1}$ and does not spread to the other infectious classes. ‘Sick’ humans are those of the class *I* who show clinical signs of the disease. ORS=0.

**Figure 4:**
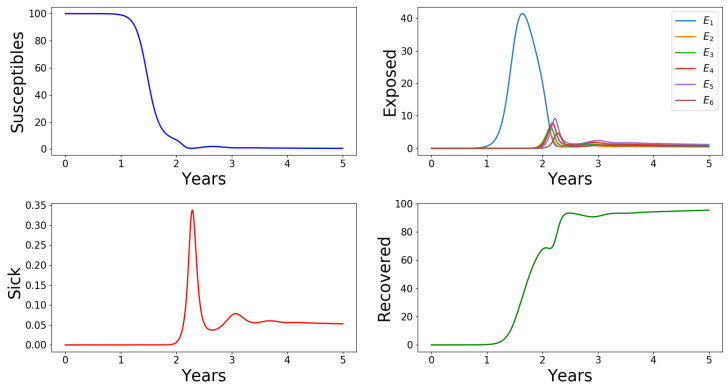
Profiles for all the variables of Model (1)–(6). The horizontal axes scale the time in days and the vertical axes scale the population sizes in percentages. The first infectious case arises in the population $E_{2}$ and is able to spread to other infectious classes so that the disease is maintained in the population. ‘Sick’ humans are those of the class *I* who show clinical signs of the disease. ORS=1:38.

The profiles for all the variables of Model (1)–(6) with initial conditions (8) and (9), are shown in Figure 3 and Figure 4, respectively. The initial conditions (8) state that the first infectious case arises in the asymptomatic class $E_{1}$, which does not naturally advance to any higher level infectious class. Thus, the total infectious population size is mostly represented by the class of $E_{1}$ humans at equilibrium (see Figure 3), whereas the asymptomatic and recovered are non-zero, and the sick humans and all other $E_{i}$${}^{\prime }$s, i∈{2,3,4,5,6} classes remain unchanged from their initial zero value. In this case, it is natural that the outbreak relative severity takes value ORS=0.

The initial conditions (9) state that the infectious case occurs in the asymptomatic class $E_{2}$, which can naturally advance to the next higher level infectious class $E_{3}$, which can advance to $E_{4}$, which can advance to $E_{5}$, which can advance to $E_{6}$, which in turn can advance to *I*. Figure 4 shows that there is a delay in the dynamics of each infectious class, including the sick and the recovered humans. In fact, the variables $E_{i}$${}^{\prime }$s, i∈{1,2,3,4,5,6}, *I* and *R* all remain very low for a minimum of approximately 1 year, then they increase sharply to their respective maximum values in a sequential order, before the decrease dramatically and stabilize at non-zero values (persistence). Similar results are obtained when the first infection case occurs in other higher level infectious classes, with the disease spreading to all other infectious classes. In this case of initial conditions (9), the outbreak relative severity ORS=1:38.

Applications of our model to actual disease data are presented in Appendix B where we fit our model to data from the DRC 1995 Ebola outbreaks (Appendix B.1) and the 2020 COVID-19 outbreak in New York State (Appendix B.2).

## 3. Discussion and Conclusion

In this paper, we introduced a mathematical model for infectious disease that includes different levels of asymptomatic latent stages of infection. While some studies have previously examined the role of asymptomatic infections co-circulating with active clinical infections during an infectious disease outbreak, we here extend this line of thinking to examine how active and circulating asymptomatic infections and associated gains in immunity can alter the disease landscape in a population, even in the absence of clinically observable cases. This initial exploration shows that the existence of asymptomatic infections can have a significant impact on the dynamics of infectious disease outbreaks, as well as drastically alter the disease landscape for future outbreaks.

These results do not alter the importance of finding and isolating clinical cases and their contacts during an outbreak response. However, if virus transmission can occur at the asymptomatic stages, there may be occasional instances of clinical cases that do not appear to have another clinical case as their origin. Such events have been observed in previous outbreaks (e.g., [34]), and, while not a definitive indicator of asymptomatic transmission, are usually attributed to failure to “capture” the appropriate index case. Importantly, the frequency of clinical cases arising solely from exposure to asymptomatic infections (and whether or not these events would alter an outbreak response plan) is certain to be highly dependent on specific characteristics of a particular outbreak and of the particular virus itself. It should be noted that the findings in the present paper pertain mainly to this model which has a number of assumptions (e.g., relationship between α,β, and λ at different *i*, and dynamics of transition between the $E_{i}$${}^{\prime }$s), and this model might be reasonable for some diseases that have an asymptomatic state (HIV, syphilis, H. pylori etc.) but not others. More study is needed to determine critical thresholds at which response plans should be modified to account for asymptomatic carriers given these characteristics.

There are available laboratory methods for many diseases that allow us to confirm whether virus is present in individuals who are not experiencing clinical symptoms, as well as test for immunity/antibodies that would indicate prior infection even if the individual did not experience symptoms. However, in an outbreak setting, conducting studies to determine which individuals have an asymptomatic infection would be a massive strain on any outbreak response and is highly unlikely to be implemented due to prioritization of locating clinical cases and limiting more common sources of the onward spread of disease. In non-outbreak situations, sampling a population to determine levels of immunity or antibodies is only slightly more feasible; in many areas, there would likely be cultural and logistical difficulties in conducting such a study (i.e., cultural: testing asymptomatic individuals for Ebola virus would likely have a large stigma attached to it; logistical: rural native ranges for a variety of emerging pathogens).

The mathematical model in this paper, for the role of asymptomatic infection in outbreaks of emerging pathogens, is generic in the sense that it can be applied to a wide range of diseases in which asymptomatic carriers play an important role. Such diseases include H1N1 influenza [35], Ebola [36], HIV [37], SARS-CoV-2 [38], hepacivirus [39], herpesvirus [40], *Mycobacterium* tuberculosis [41] and *Yersinia* pestis [42]. The model developed in this paper may be useful in studying these diseases specifically when data are available. At this time, the conclusions of the model can be viewed as hypotheses to be validated, or modified, when disease-specific experimental data are available.

The most immediate relevance of these results comes in the implications for how we expect an epidemic to develop, especially in its early stages. If a pathogen is able to sustain asymptomatic circulation (and associated levels of immunity) even in the absence of apparent clinical infections, then this inherently violates the traditional assumption that an outbreak is invading a wholly susceptible population. Such a mechanism could offer an additional explanation for why we do not see large-scale outbreaks of particular pathogens in their native ranges; but do see recurrent small outbreaks, while non-native ranges are more likely to see explosive outbreaks of clinical cases in the population, given an introduction of the pathogen. Most recently, the Ebola outbreak of 2014 may fit all the criteria for possible involvement of asymptomatic circulation. The behavior of the disease in its non-native range was substantially different from previous outbreaks [15], attempts to fit traditional SIR models suggested a role for co-emerging asymptomatic infection in West Africa [5], but to the best of our knowledge, no study has yet completed the inference to suggest that earlier outbreaks may have been intrusions of clinical cases into populations in which asymptomatic infection may already have altered the disease landscape. Extending our present model to include the movements of humans between geographic patches, the pathogen intrinsic mutations that lead to the change in the disease properties of transmission, aggressivity and mortality as a function of time, or the stochasticity of pathogen gaining endemic foothold in the community, is a logical next step in understanding this phenomenon.

## Figures and Tables

**Table 1:** Variables used in our model.

Variables	Descriptions
*S*	number of susceptible humans
$E_{i}$${}^{\prime }$s	numbers of asymptomatic (latent) humans, of various stages
*I*	number of infected humans who show clinical signs
*R*	number of recovered humans

**Table 2:** Values for the parameters used in Model (1)–(6).

Parameters	Descriptions	Values	Sources
$\alpha_{S}$, $\alpha_{i}$${}^{\prime }$s	weights of infectiousness of *S* and $E_{i}$${}^{\prime }$s by contact with *I*	0.036, $1.5^{i}$	Appendix A.2.3
*b*	rate of recruitment of humans	$1.1\times 10^{-4}\times N_{0}$ per day	Appendix A.2.1
$\beta_{I}$, $\beta_{E_{i}}$${}^{\prime }$s	rates of transmission by contact with *I* and $E_{i}$${}^{\prime }$s	0.125, $1.5^{i-(n+1)}\beta_{I}$ per day	Appendix A.2.2
$\rho_{r}$	rate of wane of immunity of *R*	$8.5\times 10^{-3}$ per day	Appendix A.2.5
$\rho_{E_{i}}$${}^{\prime }$s	rates of loss of infectiousness of $E_{i}$${}^{\prime }$s	$1.5^{i-(n+1)}\rho_{r}$ per day	Appendix A.2.5
$\lambda_{E_{i}}$${}^{\prime }$s	rates of gain of immunity of $E_{i}$${}^{\prime }$s	$1.5^{i-(n+1)}\times 0.102$ per day	Appendix A.2.4
γ	rate of removal from sick class *I*	0.167 per day	[33]
ν	rate of transition from $E_{n}$ to *I*	0.05 per day	assumed
μ	natural death rate of humans	$3.4\times 10^{-5}$ per day	Appendix A.2.1
θ	fraction of humans *I* who die	0.7	[33]
*n*	number of asymptomatic stages	6	assumed
$\tilde{n}$	number of asymptomatic stages that do not transit to higher infection stage “naturally”	1	assumed
$N_{0}$	Total initial population size	11.5 million	assumed

**Figure A1:**
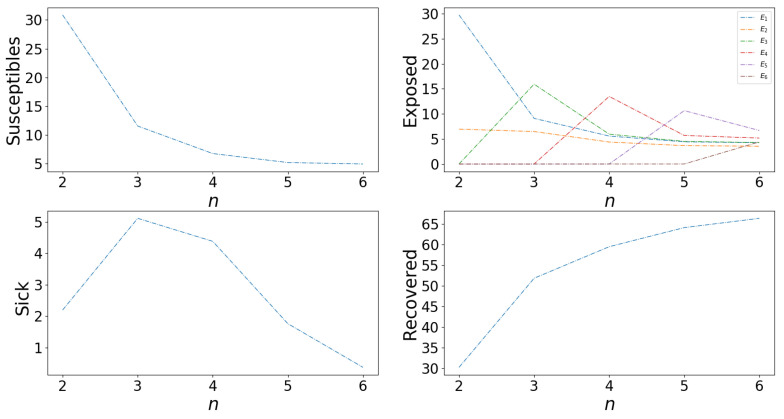
Behaviors of infectious classes relative to *n*. The horizontal axes represent the number of asymptomatic stages and the vertical axes represent the percentage of human populations. The disease can be eradicated for n≤3. There is disease persistence when n>3 which eventually leads to a branch of disease eradication for *n* large enough.

**Figure A2:**
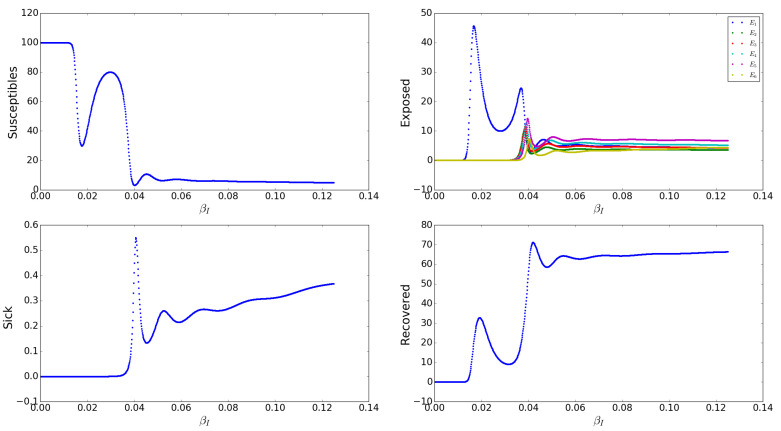
Oscillatory behaviors of infectious classes relative to the $\beta_{E_{i}}$${}^{\prime }$s (represented here by $\beta_{I}$ as in (A3)). The horizontal axes scale the baseline $\beta_{I}$ and the vertical axes represent percentages of human populations. There is a regime for the values of $\beta_{I}$ for which the disease does not persist into the population (approximately $\beta_{I}<0.014$). The disease persists into the population with oscillating profile when $0.014<\beta_{I}<0.015$; for $\beta_{I}>0.015$, the disease persists into the population stationarilly.

**Figure A3:**
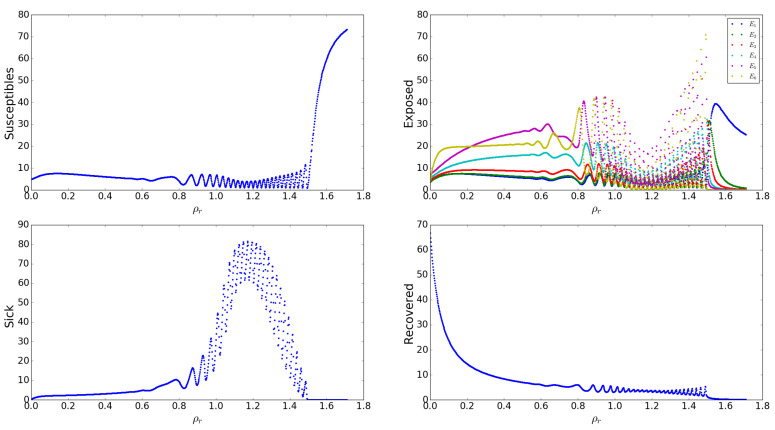
Behaviors of infectious classes relative to the $\rho_{E_{i}}$${}^{\prime }$s (represented here by $\rho_{r}$ as in (A6)). The horizontal axes scale the parameters $\rho_{r}$ and the vertical axes represent human populations. The profiles for the variables depict an endemic stationary branch followed by a chaotic behavior, then another stationary branch where the disease is eradicated.

**Figure A4:**
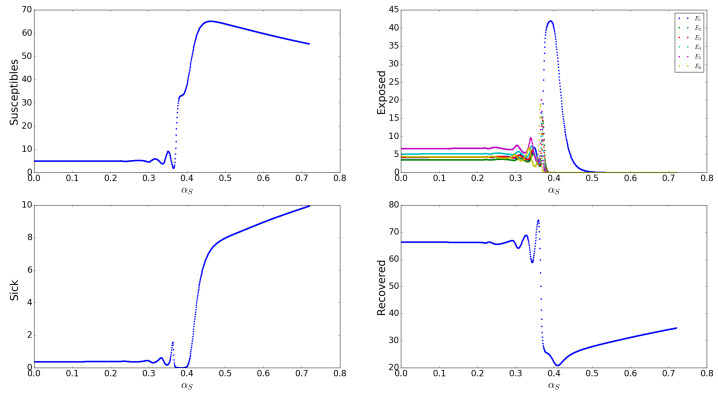
Behaviors of infectious classes relative to the $\alpha_{E_{i}}$${}^{\prime }$s (represented here by $\alpha_{S}$ as in (A4)). The horizontal axes scale the parameters $\alpha_{S}$ and the vertical axes represent percentages of human populations. The disease fatality (symptomatic cases) increases slowly ($\alpha_{S}<0.53$), then increases rapidly ($\alpha_{S}>0.53$).

**Figure A5:**
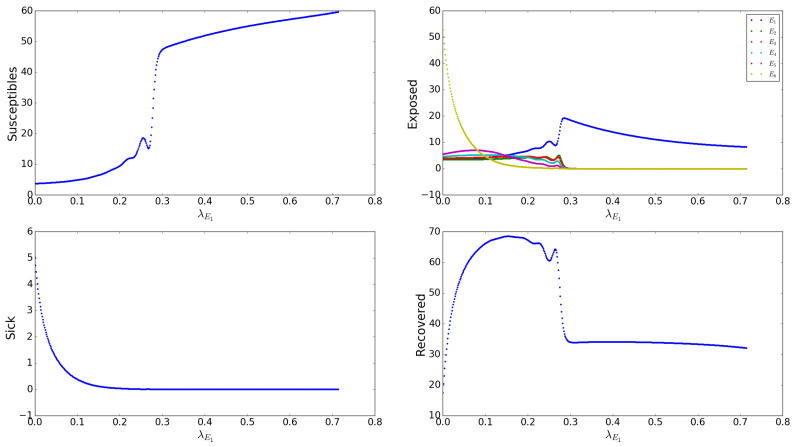
Behaviors of infectious classes relative to the λ${}^{\prime }$s (represented here by $\lambda_{E_{1}}$ as in (A5)). The horizontal axes scale the parameter $\lambda_{E_{1}}$ and the vertical axes represent the percentage of human populations. The disease persists for $\lambda_{E_{1}}$ small, then the disease is eradicated as $\lambda_{E_{1}}$ increases.

**Figure A6:**
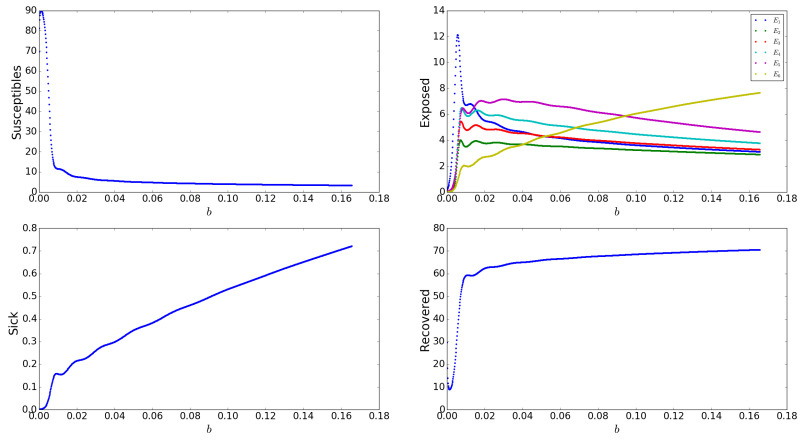
Behaviors of infectious classes relative to *b*. The horizontal axes scale the parameter *b* and the vertical axes represent percentage of human populations. The disease fatality (asymptomatic cases) mildly increases as *b* increases.

**Figure A7:**
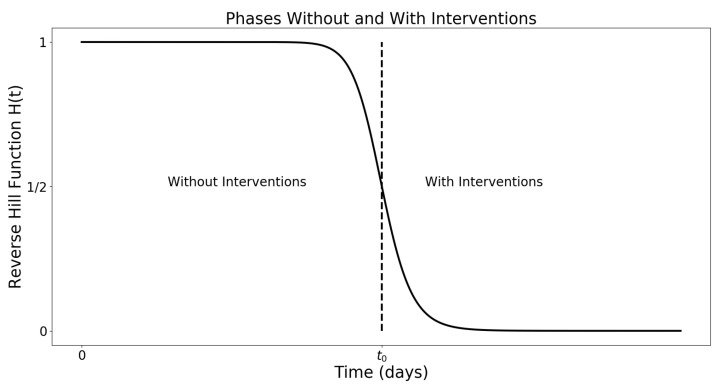
Reverse Hill Function—Phases of outbreaks without interventions and with interventions. The horizontal axis represents time in days, and the vertical axis represents the values of the reversed Hill function in (A9). The disease progresses without interventions when time $t<t_{0}$ and the ${\tilde{\beta }}_{x}=\beta_{x}$ for all *x*; the disease spreads with interventions when $t>t_{0}$ and the ${\tilde{\beta }}_{x}≃0$.

**Figure A8:**
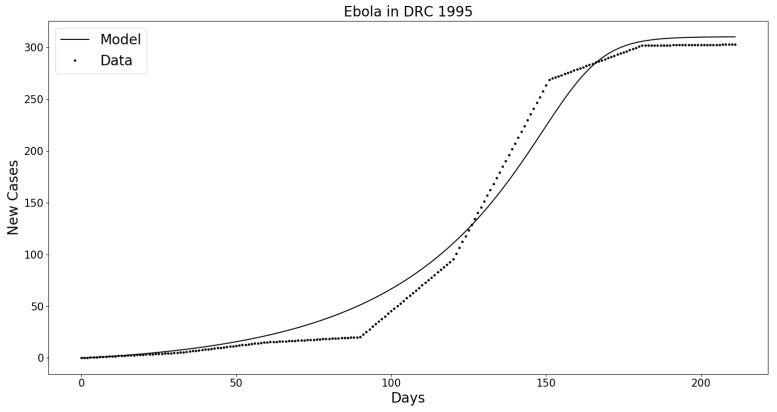
The horizontal axis represents the time in days and the vertical axis represents the cumulative number of new Ebola cases from the model simulations and actual data (data are from Figure 1 in [47]). The values of the parameters are as follows: $N_{0}=8$ millions, $\beta_{I}=0.023$ per day, $t_{0}=164$ days, $n_{H}=20$, and the values for the other parameters are as in Table 2. RE =21.04% and $R_{0}=1.49$.

**Figure A9:**
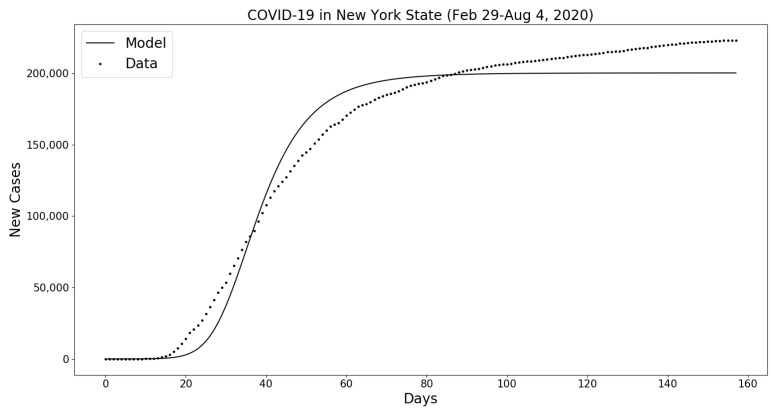
The horizontal axis represents the time in days and the vertical axis represents the cumulative number of new COVID-19 cases from the model simulations and actual data (data are from [48,49]). The values of the parameters are as follows: $\mu =3.37\times 10^{-5}$ per day [50], $b=6.4\times 10^{-4}$ pop. per day [51], $N_{0}=19.8$ millions [51], $\beta_{I}=0.066$ per day, $t_{0}=30$ days, $n_{H}=5$, and the values for the other parameters are as in Table 2. RE =23.2% and $R_{0}=12.3$.

## Appendix A. Existence of Oscillatory Outbreak Equilibrium Points

In this section, we set values for the parameters and initial conditions necessary for simulating Model (1)–(6). For illustration, we use six asymptomatic stages, n=6. The list of all parameters of the model and their numerical values is given in Table 2. Most of the parameters are taken, or estimated, from the experimental literature [43,44]. All the computations are done using Python 2.7.6.

### Appendix A.1. Local Stability Analysis of Disease-Free Equilibrium

We write the full system of equations for $n=3,\;\tilde{n}=1$, and formulate conditions for the disease eradication or persistence.

(A1) $$\begin{array}{cc} \frac{dS}{dt}= & \;b+\rho_{E_{1}}E_{1}+\rho_{r}R-\sum_{i=1}^{3}\beta_{E_{i}}E_{i}S-\alpha_{S}\beta_{I}I-\mu S\\ \frac{dE_{1}}{dt}= & \;\sum_{i=1}^{3}\beta_{E_{i}}E_{i}S+\rho_{E_{2}}E_{2}-\rho_{E_{1}}E_{1}-\sum_{i=2}^{3}\beta_{E_{i}}E_{i}E_{1}-\alpha_{E_{1}}\beta_{I}E_{1}I-\lambda_{E_{1}}E_{1}-\mu E_{1}\\ \frac{dE_{2}}{dt}= & \;\sum_{i=2}^{3}\beta_{E_{i}}E_{i}E_{1}+\rho_{E_{3}}E_{3}-\rho_{E_{2}}E_{2}-\alpha_{E_{2}}\beta_{I}E_{2}I-\beta_{E_{2}}E_{3}E_{2}-\nu_{E_{2}}E_{2}-\lambda_{E_{2}}E_{2}-\mu E_{2}\\ \frac{dE_{3}}{dt}= & \;\beta_{E_{3}}E_{3}E_{2}+\nu_{E_{2}}E_{2}-\alpha_{E_{3}}\beta_{I}E_{3}I-\rho_{E_{3}}E_{3}-\nu_{E_{3}}E_{3}-\lambda_{E_{3}}E_{3}-\mu E_{3}\\ \frac{dI}{dt}= & \;\beta_{I}\left(\alpha_{S}+\sum_{i=1}^{3}\alpha_{E_{i}}E_{i}\right)I+\nu_{E_{3}}E_{3}-\gamma I\\ \frac{dR}{dt}= & \;\gamma \left(1-\theta \right)I+\sum_{i=1}^{3}\lambda_{E_{i}}E_{i}-\rho_{r}R-\mu R \end{array}$$

The following result is easily proven by applying the next generation matrix method for computing the basic reproduction number $R_{0}$:

The disease-free equilibrium for (A1) is given by

$$DFE=\left(S^{*},E_{1}^{*},E_{2}^{*},E_{3}^{*},I^{*},R^{*}\right)=\left(b/\mu ,0,0,0,0,0\right).$$

We use the next generation matrix method to compute the basic reproduction:

(A2) $$\begin{array}{c} R_{0}=\mathrm{spectral}\_\mathrm{radius}{FV}^{-1}=\frac{b}{\mu }\times \max\left\{\frac{\beta_{E_{1}}}{\lambda_{E_{1}}+\rho_{E_{1}}+\mu },\frac{\alpha_{S}\beta_{I}}{\gamma }\right\}, \end{array}$$

where

$$F=\left(\begin{array}{cccc} b\beta_{E_{1}}/\mu & b\beta_{E_{2}}/\mu & b\beta_{E_{3}}/\mu & 0\\ 0 & 0 & 0 & 0\\ 0 & 0 & 0 & 0\\ 0 & 0 & 0 & b\alpha_{S}\beta_{I}/\mu \end{array}\right)$$

and

$$V=\left(\begin{array}{cccc} \lambda_{E_{1}}+\rho_{E_{1}}+\mu & \rho_{E_{2}} & 0 & 0\\ 0 & \lambda_{E_{2}}+\nu_{E_{2}}+\rho_{E_{2}}+\mu & -\rho_{E_{3}} & 0\\ 0 & -\nu_{E_{2}} & \lambda_{E_{3}}+\nu_{E_{3}}+\rho_{E_{3}}+\mu & 0\\ 0 & 0 & -\nu_{E_{3}} & \gamma \end{array}\right).$$

We therefore conclude that the DFE is stable when $R_{0}<1$, and unstable when $R_{0}>1$.

The expression of $R_{0}$ in (A2) shows that the disease can be controlled by minimizing the contacts of susceptible humans with symptomatic patients ($\alpha_{S}\beta_{I}/\gamma$), but also by minimizing the contacts between susceptible humans and level-1-asymptomatic humans ($\beta_{E_{1}}/\left(\lambda_{E_{1}}+\rho_{E_{1}}+\mu \right)$). Note that level-1-asymptomatic humans ($E_{1}$) are the stable carriers through which the pathogens persist in the community in absence of clinical cases.

The existence of endemic equilibrium states was investigated in Figure 3 where $I^{*}=0$, and Figure 4 where $I^{*}\neq 0$.

### Appendix A.2. Parameters Estimation

Assumptions about relationships between model parameters include the following:

(a) The rates of transmission from asymptomatic and symptomatic individuals are related (increasing one necessarily increases the other, for example).
(b) The rate of waning immunity from R is related to the rate of loss of infectiousness of $E_{i}$${}^{\prime }$s.
(c) As individuals progress through asymptomatic classes $E_{i}$, their susceptibility, transmissibility, rate of loss of infectiousness, and rate of gain of immunity all increase.

#### Appendix A.2.1. Estimate for *b* and *μ*:

According to [45], between 2008–2015, the yearly human birth rate in Africa ranged between 0.034–0.045. Similarly, within the same period of time, the yearly human death rate in Africa ranged between 0.01–0.015. We take the averages of these ranges and estimate *b* and μ as follows:

$$b=1.1\times 10^{-4}\times N_{0}\;\mathrm{per}\;\mathrm{day},\;\mathrm{and}\;\mu =3.4\times 10^{-5}\;\mathrm{per}\;\mathrm{day},$$

where $N_{0}$ is the initial total human population.

In the estimation of the parameters $\alpha_{i}$${}^{\prime }$s, $\beta_{i}$${}^{\prime }$s and $\lambda_{i}$${}^{\prime }$s, we assume that $p_{1}<p_{2}<\cdots <p_{n}$, where *p* is either α,β or λ. Note that while this ordering may be intuitive for the α${}^{\prime }$s and the $\beta^{\prime }$s, we assume that individuals who recover from higher levels of infectiousness gain larger immunity rates, the λ${}^{\prime }$s. Moreover, we assume that each parameter $p_{i}$ takes the form

$$p_{i}=p_{0}c_{p}^{i-(n+1)},\;i\in \left\{1,2,\ldots ,n\right\},$$

where $p_{0}$ is the maximum value attained by the parameter *p* and $c_{p}$ is a positive constant. Figure 2 shows the profile of *p* as the asymptomatic stage index *i* varies.

#### Appendix A.2.2. Estimate for *β_I_* and $\beta_{E_{i}}$, *i* = 1,2,…,*n*:

Siewe et al. [46] estimated the range for the rate of transmission of Evd in DRC to be 0.0798–0.13 per day; we take $\beta_{I}=0.125\;\mathrm{per}\;\mathrm{day}$. Since the force of infection of asymptomatic humans $E_{i}$${}^{\prime }$s increases as the index *i* increases, we assume that

(A3) $$\begin{array}{c} \beta_{E_{i}}=c_{\beta }^{i-(n+1)}\beta_{I}=c_{\beta }^{i-(n+1)}\times 0.125\;\mathrm{per}\;\mathrm{day},\;i=1,2,\ldots ,n, \end{array}$$

where *n* is the number of asymptomatic levels considered.

#### Appendix A.2.3. Estimate for *α_S_* and $\alpha_{E_{i}}$, _i_ = 1,2,…,*n*:

We take the value of $\alpha_{S}$ in the term $\alpha_{S}\beta_{I}SI$ in Equation (2.1) to be $\alpha_{S}=0.036$. Now, we assume that $\alpha_{E_{1}}<\alpha_{E_{2}}<\cdots <\alpha_{E_{n}}$, and take

(A4) $$\begin{array}{c} \alpha_{E_{i}}=c_{\alpha }^{i-(n+1)}\alpha_{S}=c_{\alpha }^{i-(n+1)}\times 0.036,\;i=1,2,\ldots ,n, \end{array}$$

#### Appendix A.2.4. Estimate for $\lambda_{E_{i}}$, *i* = 1,2,…,*n*:

We assume that the rates at which the asymptomatic humans gain immunity is larger as the level of infectiousness increases. That is, we assume that $\lambda_{E_{n}}>\cdots >\lambda_{E_{2}}>\lambda_{E_{1}}$ and take

(A5) $$\begin{array}{c} \lambda_{E_{i}}=c_{\lambda }^{i-(n+1)}\times 0.0102,\;i=1,2,\ldots ,n. \end{array}$$

#### Appendix A.2.5. Estimate for $\rho_{E_{i}}$, *i* = 1,2,…,*n*:

We assume that the rates at which the asymptomatic humans lose infectious severity is larger as the level of infectiousness increases. That is, we assume that $\rho_{E_{n}}>\cdots >\rho_{E_{2}}>\rho_{E_{1}}$ and take

(A6) $$\begin{array}{c} \rho_{E_{i}}=c_{\rho }^{i-(n+1)}\times 3.8\times 10^{-4},\;i=1,2,\ldots ,n. \end{array}$$

In the sequel, we take $c_{p}=1.5$, where p∈{α,β,λ,ρ}.

#### Appendix A.2.6. Effects of Number of Asymptomatic Cases, *n*

Since asymptomatic infection in reality does not occur on a discretized scale, increasing the number of asymptomatic bins can be interpreted as increasing the average human tolerance for viral load before clinical signs begin to show. We vary *n* between 2–6, with $\tilde{n}=1$, while we keep the values for all other parameters fixed at their baselines in Table 2. Figure A1 shows the change in the end points of the levels for each variable in Model (2.1)–(2.6), with respect to *n*. We see that the disease can be eradicated for n≤3. There is disease persistence when n>3 which eventually leads to a branch of disease eradication for *n* large enough.

#### Appendix A.2.7. Effects of the Asymptomatic Transmission Rates $\beta_{E_{i}{}^{\prime }}$s

We vary $\beta_{I}$ in (A3) between 0–0.03 and record the values of the variables in (2.1)–(2.6) after fifteen years of control. We see in Figure A2 that there is a regime for the values of $\beta_{I}$ for which the disease does not persist into the population (approximately $\beta_{I}<0.014$). The disease persists into the population with oscillating behavior when $0.014<\beta_{I}<0.015$. For values of $\beta_{I}>0.015$, the disease persists in the population stationarilly.

#### Appendix A.2.8. Effects of the Loss/Recovery of Asymptomatic Infection Rates $\rho_{E_{i}{}^{\prime }}$s

We vary $\rho_{r}$ in (A6) between 0–8 and record the values of the variables in (2.1)–(2.6) after fifteen years of control. We see in Figure A3 that increasing the rate of loosing immunity causes oscillations in equilibrium points of the system. In fact, the disease depicts an endemic stationary branch followed by a chaotic behavior, then another stationary branch where the disease is eradicated.

#### Appendix A.2.9. Effects of the Clinical Transmission Rates $\alpha_{E_{i}{}^{\prime }}$s

We vary $\alpha_{S}$ in (A4) between 0–0.7 and record the values of the variables in (2.1)–(2.6) after fifteen years of control. Figure A4 shows that he disease fatality (symptomatic cases) increases slowly ($\alpha_{S}<0.53$), then increases rapidly ($\alpha_{S}>0.53$).

#### Appendix A.2.10. Effects of the Gain of Immunity Rates by Asymptomatic Humans, $\lambda_{E_{i}{}^{\prime }}$s

We vary $\lambda_{E_{1}}$ in (A5) between 0–0.7 and record the values of the variables in (2.1)–(2.6) after fifteen years of control. Figure A5 shows that the disease persists for $\lambda_{E_{1}}$ small, then the disease is eradicated as $\lambda_{E_{1}}$ increases. Interestingly, the population becomes mainly susceptible for $\lambda_{E_{1}}$ very large.

#### Appendix A.2.11. Effects of Birth Rate, *b*

We vary *b* between 0–0.0035 and record the values of the variables in (2.1)–(2.6) after fifteen years of control. Figure A6 shows that the disease fatality (asymptomatic cases) mildly increases as *b* increases.

## Appendix B. Ebola (DRC 1995) and COVID-19 (New York 2020)

In this section, we compare the simulations of our parameterized model with $n=3,\;\tilde{n}=1$ in (A1) with actual data from a previous Ebola outbreak in DRC (Figure 1 in [47]), and COVID-19 in New York (29 February–4 August 2020 [48,49]) and capture the number of new cases from these outbreaks, defined by

(A7) $$\begin{array}{c} \mathrm{new}\_\mathrm{cases}\left(t\right)=\int_{0}^{t}\left(\sum_{i=1}^{3}\beta_{E_{i}}E_{i}\left(\tau \right)+\alpha_{S}\beta_{I}I\left(\tau \right)\right)S\left(\tau \right)d\tau . \end{array}$$

We measure the goodness of fit by computing the basic reproduction number $R_{0}$ in (A2) and the data-simulations relative error (RE) as follows:

(A8) $$\begin{array}{c} \mathrm{RE}=\frac{\sum {\left(\mathrm{simulations}-\mathrm{data}\right)}^{2}}{\sum {\left(\mathrm{data}\right)}^{2}}\left(\times 100\%\right). \end{array}$$

Note that the outbreaks evolve in two parts: the increasing phase of the infection where the number of new cases increases exponentially, and the decreasing phase where the number of new infectious cases drops. These two phases of of the outbreaks are represented in our model by adjusting the rates of transmission by contact with infectious humans to be time dependent, and we take the following reverse Hill function which represents the time of infection without intervention and the time with intervention:

(A9) $$\begin{array}{c} {\tilde{\beta }}_{I}\left(t\right)=\beta_{I}H\left(t\right),\;\mathrm{where}\;H\left(t\right)=\frac{t^{-n_{H}}}{t^{-n_{H}}+t_{0}^{-n_{H}}}; \end{array}$$

$\beta_{I}$ is outbreak-specific and the corresponding ${\tilde{\beta }}_{E_{i}}$${}^{\prime }$s are defined as in (A3).

### Appendix B.1. Ebola in the Democratic Republic of Congo (DRC) 1995

To capture the number of new Ebola cases for the DRC 1995, we take the value of $N_{0}$ to be the approximate total population size in the DRC province of Bandudu ($N_{0}=8$ millions). Figure A8 shows our model simulations and actual data for the DRC 1995 Ebola outbreak, where RE = 21.04% which indicates good fit, and the basic reproduction number $R_{0}=1.49$ which compares well with those in the literature ($R_{0}=1.83$ in [26] and $R_{0}=1.53$ in [46]).

### Appendix B.2. COVID-19 in New York State (February 29–4 August 2020)

We show our model simulations and actual data for the New York 2020 Covid-19 outbreaks in Figure A9. The cases were reported between 29 February (first reported case) and 4 August 2020. We obtain a fit of RE = 23.2% and $R_{0}=12.3$. Our model does not capture the resurgence of the disease after the first three months of outbreak. In fact, we see that while our model simulations are represented by a plateau due to the assumption of ‘perfect’ intervention or prevention methods (see Figure A7), the actual data continue to increase at smaller rate beyond the first three months of outbreak.
